# The effect of radiation exposure on multidrug resistance: *in vitro* and *in vivo* studies using non-small lung cancer cells

**DOI:** 10.1186/s13550-015-0091-5

**Published:** 2015-03-17

**Authors:** Shohei Kanno, Keita Utsunomiya, Yumiko Kono, Noboru Tanigawa, Satoshi Sawada

**Affiliations:** Department of Radiology, Kansai Medical University, 2-5-1 Shinmachi, Hirakata, 573-1010 Osaka Japan

**Keywords:** ^99m^Tc MIBI, Doxorubicin, Irradiation, MDR, Non-small cell lung cancer

## Abstract

**Background:**

Technetium-99m methoxyisobutylisonitrile (Tc MIBI) is a substrate with the same uptake kinetics as doxorubicin. Multidrug resistance (MDR) is a mechanism that impedes chemotherapy of non-small cell lung cancer (NSCLC). We examined the effect of radiation exposure on MDR in NSCLC and the synergy between an MDR modulator, GG918, and radiation, using ^99m^Tc MIBI *in vitro* and doxorubicin *in vivo*.

**Methods:**

*In vitro* NSCLC cells (H1299) were exposed to radiation (3-, 6-, and 9-Gy-irradiated groups) alongside a not-irradiated (0 Gy) group. Technetium-99 metastable methoxyisobutylisonitrile (^99m^Tc MIBI) was administered to cell suspensions at 48 h after irradiation. Cell radioactivity was measured, and *C*_in_/*C*_out_ ratios were calculated and compared. NSCLC cells were also subcutaneously transplanted into the left thigh of nude mice, which were subsequently raised for 2 weeks. Two groups of mice were used: mice exposed to irradiation (9-Gy-irradiated) and those that were not (not-irradiated). Doxorubicin was administered through the caudal vein at 48 h after the irradiation. Using an *in vivo* imaging system, intratumoural photon counts were measured. To determine the synergy between the MDR modulator and 3- or 6-Gy irradiation, the final GG918 concentration was determined: 0.1 μM (N-H, 3-H, and 6-H groups), 0.001 μM (N-L, 3-L, and 6-L groups), and 0 μM (N-0, 3-0, and 6-0 groups). *C*_in_/*C*_out_ ratios were calculated and compared among the groups.

**Results:**

*C*_in_/*C*_out_ after 6- or 9-Gy irradiation was significantly higher than that of the not-irradiated group (0 Gy). *In vivo*, fluorescence photon counts were significantly higher in the tumours of 9-Gy-irradiated mice, up to 270 min after administration of doxorubicin, as compared to the not-irradiated mice. The *C*_in_/*C*_out_ ratio in the N-H, 3-H, and 6-H groups was significantly higher than that in the N-0, 3-0, and 6-0 groups. There was no significant difference between *C*_in_/*C*_out_ in the N-L group and that of the N-0 group. However, the *C*_in_/*C*_out_ ratio in the 3-L and 6-L groups was significantly higher than that in the 3-0 and 6-0 groups.

**Conclusions:**

Irradiation decreased MDR in NSCLC cells. In combination with a low-dose MDR modulator, GG918, MDR transport function was synergistically reduced 48 h post-irradiation.

## Background

Non-small cell lung cancer (NSCLC) accounts for more than 80% of diagnosed lung cancer cases and is often detected at an advanced stage, rendering it extremely difficult to treat with current therapeutic regimes. Lung cancer that is detected in the advanced stage may be treated with molecular-targeted drugs; however, it is commonly treated with chemoradiotherapy [1]. For chemotherapy, the current practice is to deliver several anticancer drugs with different mechanisms of action, thereby reducing the dose of each drug required, thus, decreasing the occurrence of side effects, as well as benefitting from synergistic tumour reduction effects. Thus, combination therapy is commonly used as a treatment regimen for lung cancer.

On the other hand, radiotherapy depends on the sensitivity of specific tumour cells to radiation. The effectiveness of hyperfractionated radiation therapy, i.e., irradiation over optimal intervals, targeting the most sensitive stages of the tumour cell cycle, has also been investigated. The goal of established chemoradiotherapy is to achieve tumour volume reduction, whereas novel combinations of chemotherapy and radiotherapy aim to reduce both tumour volume as well as the occurrence of side effects. However, combination therapies are often discontinued because of the side effects of both irradiation and anticancer drugs.

Furthermore, there is a growing concern that treatment efficacy may not be sufficient because of multidrug resistance (MDR) [2]. The overexpression of P-glycoprotein (Pgp) and breast cancer-resistant protein (BCRP) in the membranes of tumour cells plays a major role in the development of MDR [3,4]. Cancer cells bearing the MDR phenotype are capable of eliminating anticancer drugs, such as cisplatin or doxorubicin, by using Pgp and BCRP as an efflux channel protein, thereby diminishing their chemotherapeutic actions [4].

^99m^Tc MIBI, used for myocardial perfusion scintigraphy and imaging of the parathyroid gland, is a substrate of Pgp and is known to have similar tumour cell kinetics to cisplatin and doxorubicin [5]. In addition, MDR modulators (e.g., verapamil, GG918, PSC833, and cyclosporin A) reduce the transporter function of Pgp and BCRP [6,7]. These modulators bind to Pgp and BCRP, thereby preventing these membrane-bound protein channels from transporting anticancer drugs out of tumour cells. However, to achieve this effect, a high dose of such modulators must be administered [8,9]. Consequently, these modulators have not been used in combination with any chemoradiotherapeutic or chemotherapeutic regimen, due to concerns regarding their toxicity.

In this study, NSCLC cells (*in vitro*) and tumours (*in vivo*) with an MDR phenotype were exposed to varying doses of radiation, as well as ^99m^Tc MIBI and doxorubicin, respectively, to investigate the effect of radiation on MDR and MDR modulators.

## Methods

### Ethical consideration

In this study, the institutional and national guide for the care and use of laboratory animals was followed and the protocols used were approved by the Institutional Review Board of Kansai Medical University.

### Reagents

^99m^Tc MIBI (Cardiolite; Fujifilm RI Pharma., Co., Ltd., Tokyo, Japan) and doxorubicin (Adriamycin; Kyowa Hakko Kirin Co., Ltd., Tokyo, Japan) were used as tracers. A stock solution of ^99m^Tc MIBI was prepared by mixing 10 MBq of ^99m^Tc MIBI with 2 mL of saline solution; this solution was then used in the *in vitro* and *in vivo* experiments [10,11]. A stock solution of doxorubicin was prepared by mixing 1 mg of doxorubicin with 1 mL of saline solution. This solution was then used in the *in vivo* experiment. A stock solution of MDR modulator was prepared from an acridone carboxamide derivative, GG918, (N-{4-(2-(1,2,3,4-tetrahydro-6,7-dimethoxy-2-isoquinolinyl)-ethyl)-phenyl}-9,10-dihydro-5-methoxy-9-oxo-4-acridine carboxamide; Elacridar; MedChem Express, LLC, Monmouth Junction, NJ, USA) [7,12].

### Cell culture and xenografts

*In vivo* and *in vitro* experiments were conducted using NSCLC cells (H1299), transfected with a wild-type P53-encoding gene, which was supplied by Hideki Matsumoto, Ph.D., Oncology, Biomedical Imaging Research Centre, Fukui University, Japan [13]. ICR nude mice (Crlj: *CD1-Foxn1nu*) were used for xenografting (Charles River Laboratories, Wilmington, MA, USA) [14].

For the *in vitro* study, H1299 cells were cultured as monolayers in Dulbecco’s alpha-MEM medium (D-MEM; Sigma-Aldrich, St. Louis, MO, USA) supplemented with 10% (*v*/*v*) foetal bovine serum (Equitech-Bio, Kerrville, TX, USA), 50 μg/mL streptomycin, and 200 μg/mL geneticin and were incubated at 37°C in 95% air and 5% CO_2_. Prior to culturing, the cells were subjected to 3-, 6-, and 9-Gy (irradiated groups) gamma irradiation (Gammacell 40 Exactor, Nordion International, Ottawa, Canada). Cells from the irradiated groups and the not-irradiated group were cultured for 48 h as previously described. H1299 cells were trypsinized and centrifuged, and each cell suspension was diluted to a density of 0.5 to 1.0 × 10^6^ cells/mL [10,11].

For the *in vivo* study, H1299 cells were cultured as previously described, trypsinized, and then centrifuged four times with Dulbecco’s phosphate-buffered saline (D-PBS; Gibco; Invitrogen Co., Ltd, Carlsbad, CA, USA) at 4°C, after which cells were resuspended in D-PBS. We transplanted 5.0 × 10^7^ cells into the left thighs of mice, which were then raised for 2 weeks to allow tumour formation [12,14,15]. After 2 weeks, the tumour volume (mm^3^) was calculated; tumours ≥20 mm^3^ were used for further study. Xenografts were subjected to 9-Gy gamma irradiation (Gammacell 40 Exactor, Nordion International, Ottawa, Canada), incubated for 48 h, and then used for experimentation.

### Expression of transporters in H1299 cells

H1299 cells were smeared onto silane-coated slides (No. 5136, MUTO, Co.. Ltd., Tokyo, Japan) and fixed in 100% ethanol. The cells were incubated in 0.3% H_2_O_2_ in 0.05 M D-PBS for 15 min at room temperature and then washed three times with 0.05 M PBS for 3 min. Afterwards, the cells were blocked with 5% skim milk (*v*/*v*) and 0.15% H_2_O_2_ (*v*/*v*) in de-ionized water for 20 min at room temperature. Primary antibodies against Pgp and BCRP were then added and allowed to react with the cells overnight at room temperature. Transporters were detected using a 1/500 dilution of anti-BCRP/ABCG2 antibody (ab72788, Abcam, Co., Ltd., Cambridge, MA, USA) or anti-Pgp monoclonal antibody (4E3.16, #517308, Calbiochem, Co., Ltd., San Diego, CA, USA). Isotype control was conducted using mouse IgG1 (ICIGG1; ab91353, Abcam, Co., Ltd., Cambridge, MA, USA) and mouse control IgG2a [MOPC-173] - ChIP Grade (ab18413).

After washing with 0.05 M PBS, the cells were incubated in 0.05 M D-PBS containing peroxidase-labelled polymer conjugated to goat anti-mouse immunoglobulin for 30 min. After washing with 0.05 M D-PBS, the slides were stained with 3-3′-diaminobenzidine-4HCl (DAB) for 1 min. Finally, the slides were washed in 0.05 M PBS and stained with 50% Mayer’s haematoxylin on the cover slide for 1 min [15].

To assess the quality of Pgp and BCRP expression, Allred scores were used; the proportion score (PS); the percentage of cells that were stained by immunocytochemistry (on a scale of 0 to 5) and the intensity score (IS); the intensity of the staining (on a scale of 0 to 3), for a total possible score of 8. Total scores were compared between the 9-Gy-irradiated and not-irradiated tumour cells [16].

### Cellular accumulation of ^99m^Tc MIBI

To determine cellular transport activity, cell suspensions (7.0 mL) were thoroughly mixed and incubated in a 37°C water bath. Approximately 100 μL of ^99m^Tc MIBI was then added to the cell suspensions. To evaluate intracellular accumulation, 300 μL aliquots were collected in duplicate 1, 15, 30, 45, and 60 min after ^99m^Tc MIBI administration. The aliquots were transferred to 1.5-mL microcentrifuge tubes containing 1.0 mL of ice-cold saline and centrifuged at 14,000 rpm for 2 min; the supernatants were removed, and the cells washed gently with 0.5 mL of ice-cold saline solution. Radioactivity in the cell pellets was measured at 90 to 190 keV using a gamma-counter (WIZARD**™** 3” Model 1480 Automatic Gamma Counter, PerkinElmer Life Sciences, Co., Ltd., Waltham, MA, USA). The accumulation ratio (*C*_in_/*C*_out_) was calculated as the ratio of intracellular to extracellular radioactivity in an equal volume of supernatant medium, using an independent measurement of cell volume as described previously [11]. The time course of ^99m^Tc MIBI accumulation was measured in the not-irradiated group and in the 3-, 6-, and 9-Gy-irradiated groups [10,11].

### Accumulation of doxorubicin in tumours

**Figure 1:**
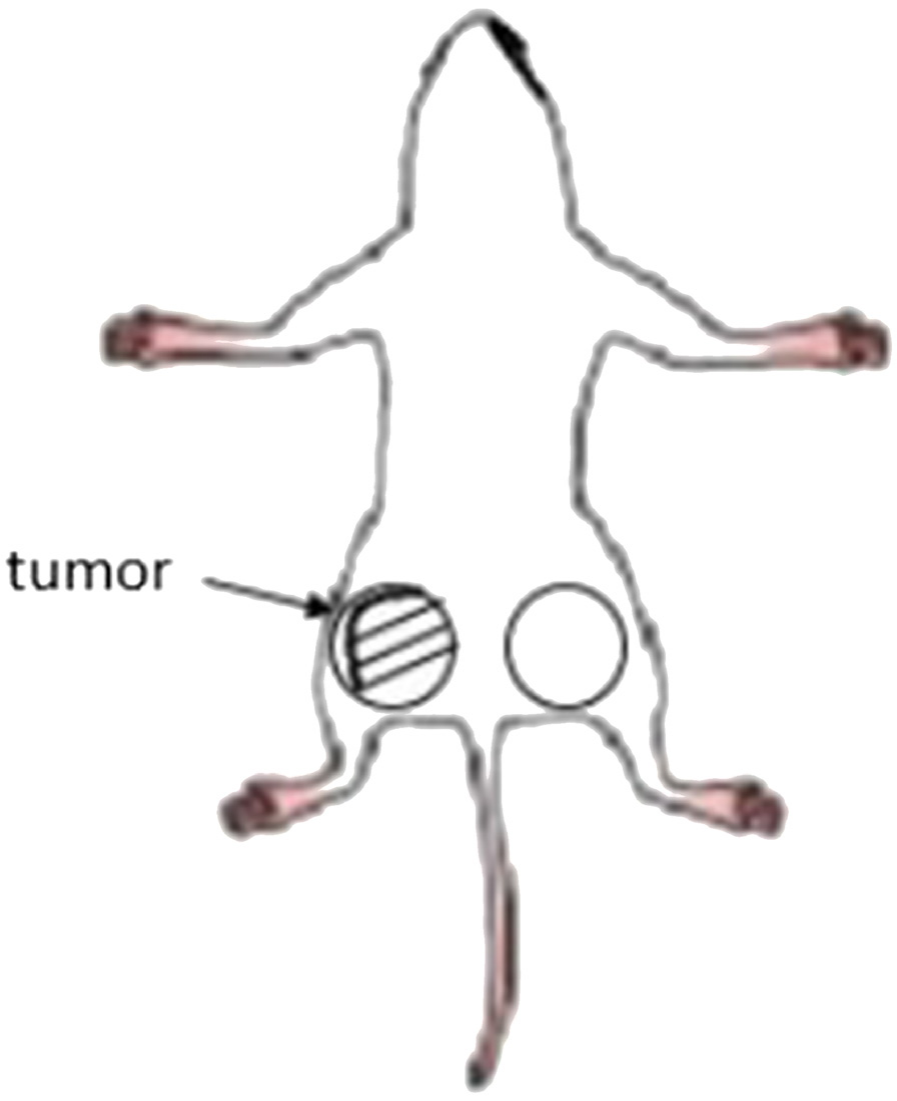
**Region of interest (ROI) setting of the xenograft**. The ROIs were set to the contralateral site of the affected area, using the same size as the tumour site in the thigh.

Doxorubicin (stock solution, 100 μL) was administered via the caudal vein to the not-irradiated mice and the 9-Gy-irradiated mice at 48 h after irradiation for *in vivo* fluorescence imaging [17]. Fluorescence (photons·s^−1^·cm^−2^) was measured, to evaluate the accumulation of doxorubicin (Figure 1), in the xenografted tumour thigh region and in the bilaterally symmetrical control thigh region using an *in vivo* imaging system (IVIS 200, Caliper Life Science Inc., Hopkinton, MA, USA) equipped with air-cooled argon lasers (emission at 488 nm) and a 575-nm band pass filter [11,15]. Changes in doxorubicin accumulation in the ROIs were detected by fluorescence imaging over time. Washout rate was calculated using the following equation:

$$ \begin{array}{l}\mathrm{Doxorubicin}\ \mathrm{washout}\ \mathrm{rate}\ \left(\%\right)=\left\{\right(\mathrm{Fluorescence}\ \mathrm{in}\ \mathrm{the}\ \mathrm{tumour}\ \mathrm{at}\ 10\  \min\ \mathrm{after}\ \\ {}\mathrm{doxorubicin}\ \mathrm{administration}-\mathrm{Fluorescence}\ \mathrm{at}\ 60,\ 120,\ \mathrm{or}\ 270\  \min\ \mathrm{after}\ \mathrm{doxorubicin}\ \\ {}\mathrm{administration}\Big)/\mathrm{Fluorescence}\ \mathrm{at}\ 10\  \min\ \mathrm{after}\ \mathrm{doxorubicin}\ \mathrm{administration}\Big\}\times 100.\end{array} $$

### Effects of GG918 on ^99m^Tc MIBI accumulation in cells after irradiation

H1299 cells were subjected to 3- and 6-Gy gamma irradiation. Irradiated cells and not-irradiated cells were separately cultured in tissue culture flasks. H1299 cells were trypsinized and centrifuged to prepare 7 mL of single-cell suspensions in fresh media with a density of 0.5 to 1.0 × 10^6^ cells/mL. GG918 was added to a final concentration of 0, 0.001, and 0.1 μM to the irradiated and not-irradiated cell suspensions at 5 min before addition of ^99m^Tc MIBI. In the not-irradiated group, the cell suspensions to which 0, 0.001, and 0.1 μM of GG918 were added were defined as the N-0 group, the N-L group, and the N-H group, respectively. In the 3- or 6-Gy-irradiated groups, the cells to which 0, 0.001, and 0.1 μM of GG918 were added were defined as the 3-0 group or 6-0 group, the 3-L group or 6-L group, and the 3-H group or 6-H group, respectively.

Radioactivity was measured using a gamma counter 30 min after the administration of ^99m^Tc MIBI, and the ^99m^Tc MIBI cellular accumulation ratio (*C*_in_/*C*_out_) was calculated. The accumulation of ^99m^Tc MIBI was compared between the N-H, N-L, and N-0 groups and both the 3-H, 3-L, and 3-0 groups and the 6-H, 6-L, and 6-0 groups.

### Statistical analysis

The samples used in the *in vitro* experiments were measured in duplicate. The data were expressed as the mean ± standard error (SE). The Kruskal-Wallis test and a *post hoc* multiple comparison test were conducted using Fisher’s protected least significant difference (PLSD) test. A *P* value less than 0.05 was considered statistically significant.

## Results

### Characterization of transporters in H1299 cells

**Figure 2:**
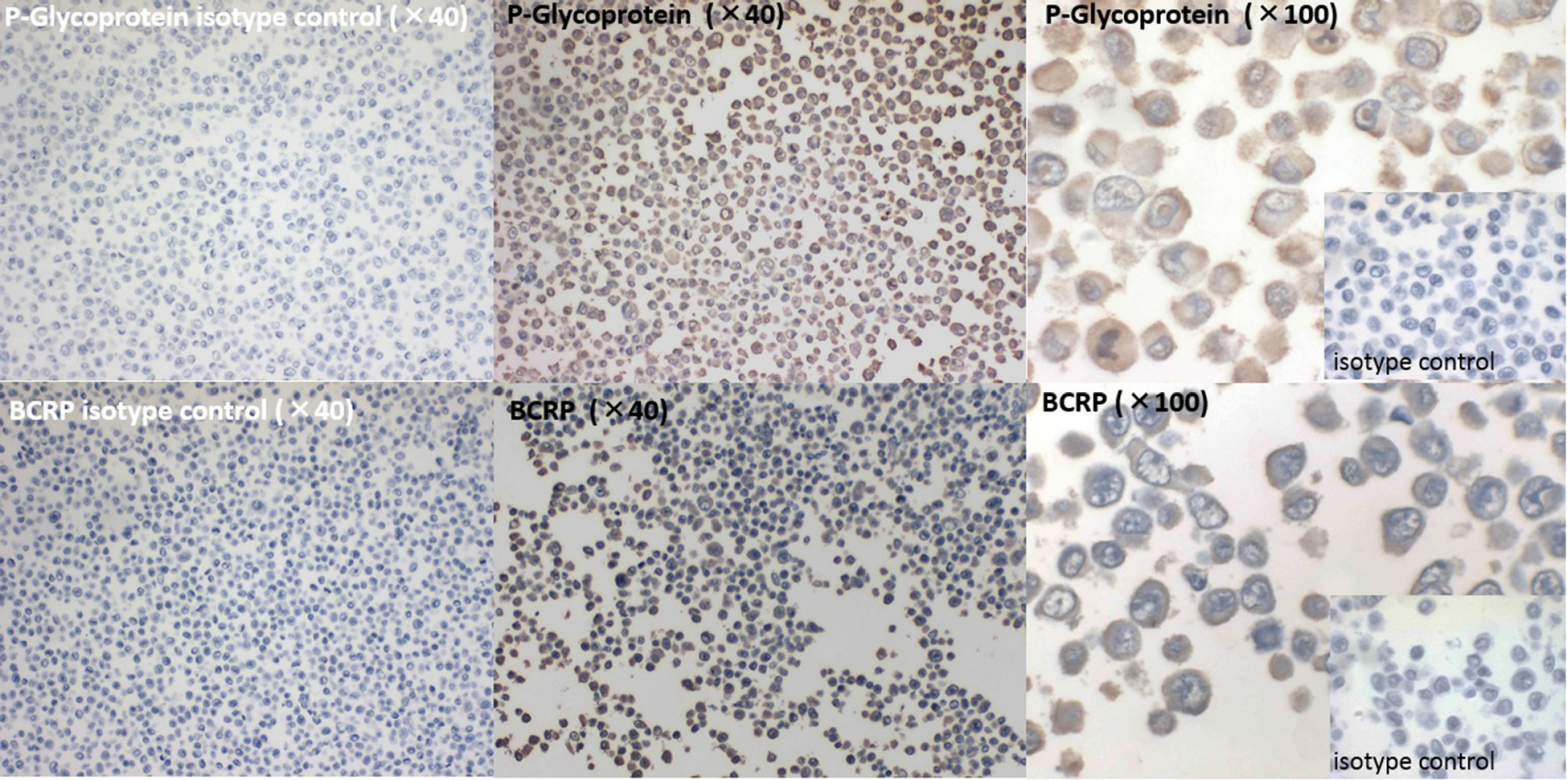
**The immunocytochemistrical expression of Pgp and BCRP.** The immunocytochemistrical expression of Pgp and BCRP was positive, the proportion score (PS) 5/5 + the intensity score (IS) 3/3 = total score 8/8, localizing these proteins to the cell membrane of the not-irradiated tumour cells.

**Figure 3:**
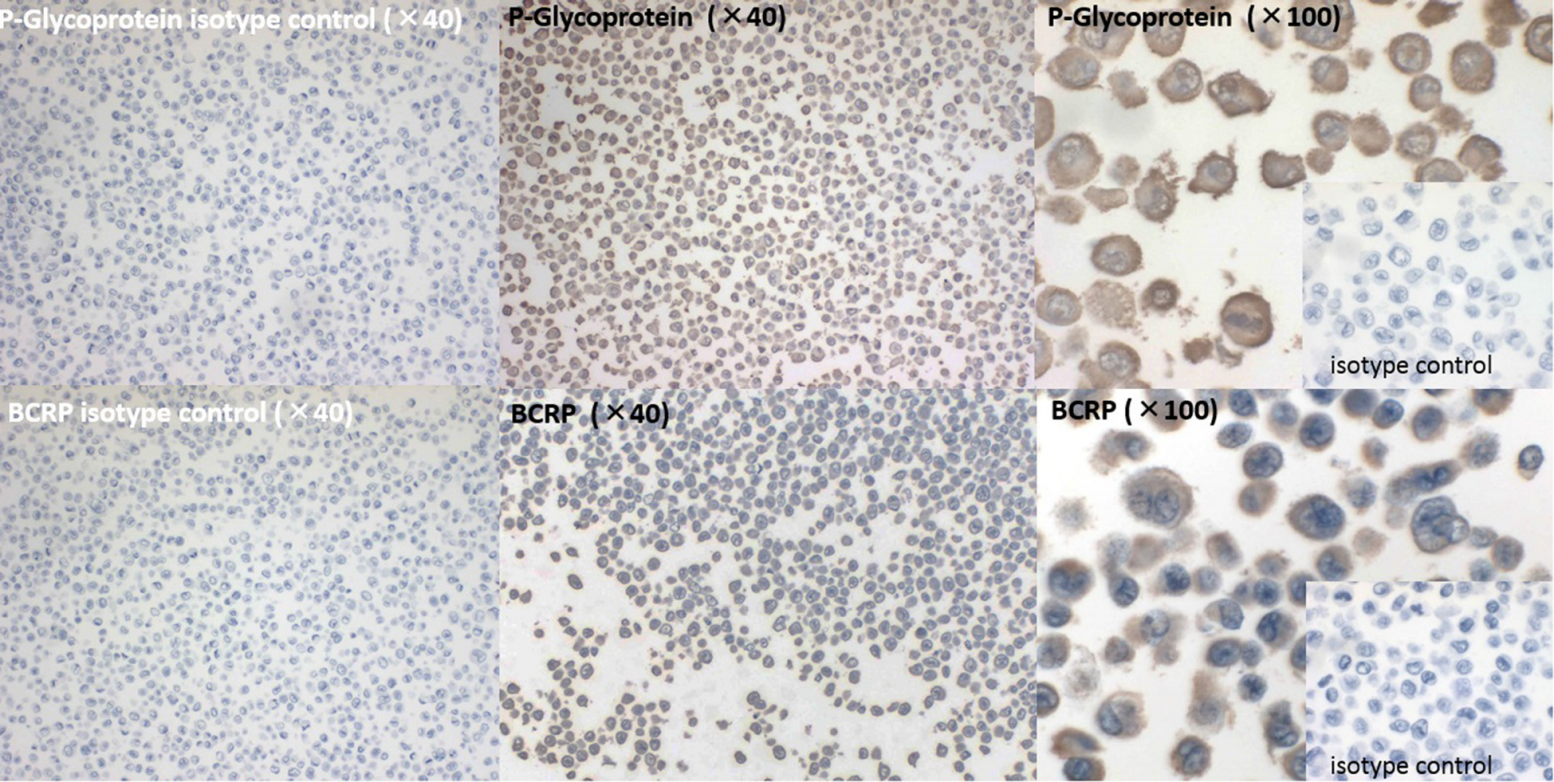
**The immunocytochemistrical expression of Pgp and BCRP in tumour cells, at 48 h after 9**-**Gy irradiation.** The immunocytochemistrical expression of Pgp and BCRP was positive, PS 5/5 + IS 3/3 = total score 8/8, localizing these proteins to the cell membrane of the 9-Gy-irradiated tumour cells. There was no difference in the expression of Pgp and BCRP between cells before and after 9-Gy irradiation.

Immunodetection of Pgp and BCRP was positive, with these proteins localized in the membranes of H1299 cells (Figures 2 and 3). In the not-irradiated cells shown in Figure 2, the expression of Pgp and BCRP were as follows: PS 5 + IS 3 = 8. There was no change after 9-Gy irradiation, followed by 48 h of culturing, as shown for the cells in Figure 3, with both Pgp and BCRP being PS 5 + IS 3 = 8.

### Cellular accumulation of ^99m^Tc MIBI after irradiation

**Figure 4:**
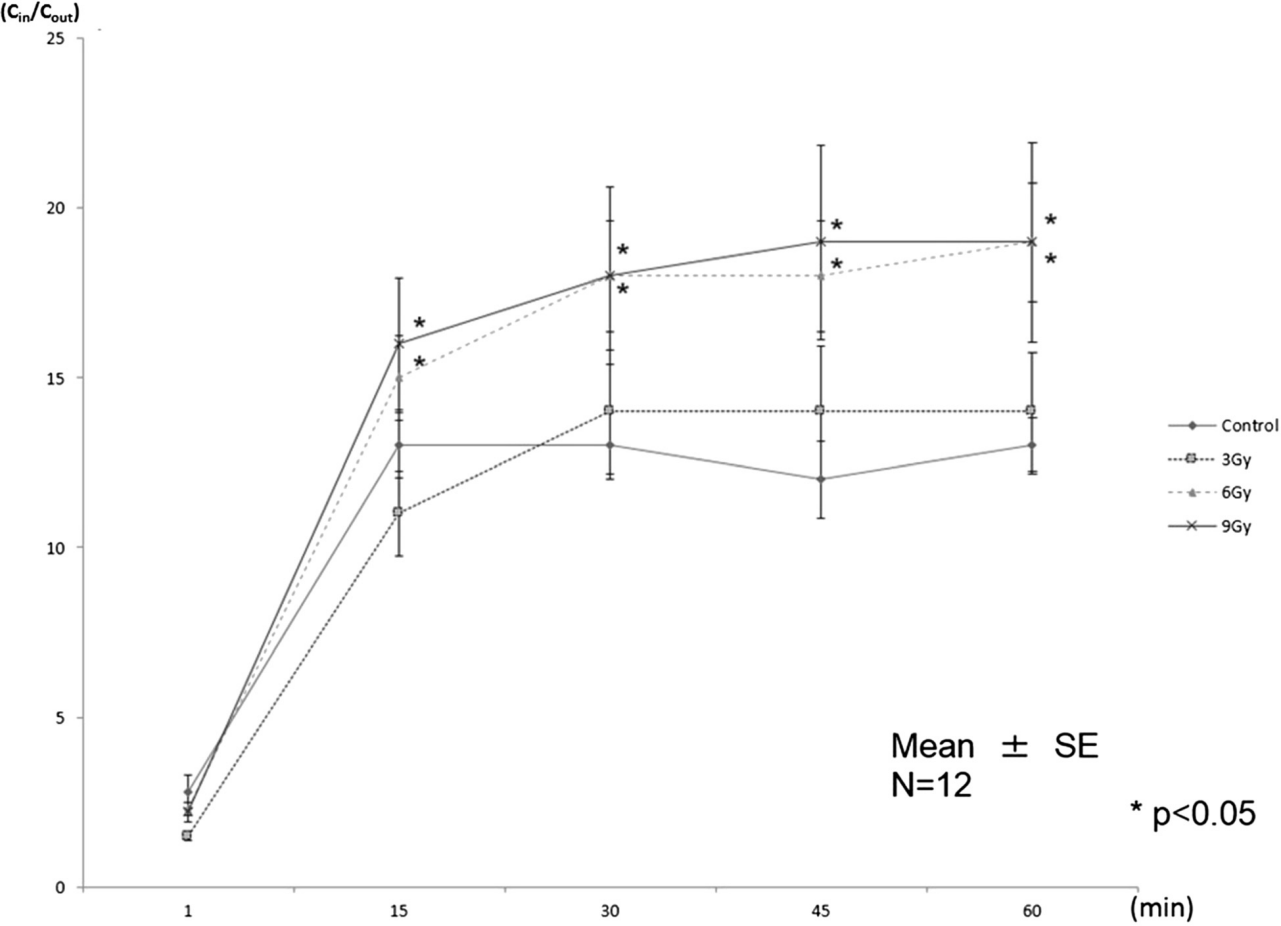
^**99m**^ **Tc MIBI accumulation study.** Compared to the not-irradiated group, the cellular uptake of ^99m^Tc MIBI increased statistically significantly in the 6-Gy group and the 9-Gy group.

The cellular accumulation of ^99m^Tc MIBI is shown in Figure 4. *C*_in_/*C*_out_ increased after approximately 30 min. *C*_in_/*C*_out_ in all groups reached a plateau within 60 min. *C*_in_/*C*_out_ in the 6-Gy-irradiated and 9-Gy-irradiated groups was higher than that in the 3-Gy-irradiated group and the not-irradiated group (*P* < 0.05). There was no significant difference observed between the 3-Gy-irradiated group and the not-irradiated group.

### Doxorubicin accumulation in tumours after irradiation

**Figure 5:**
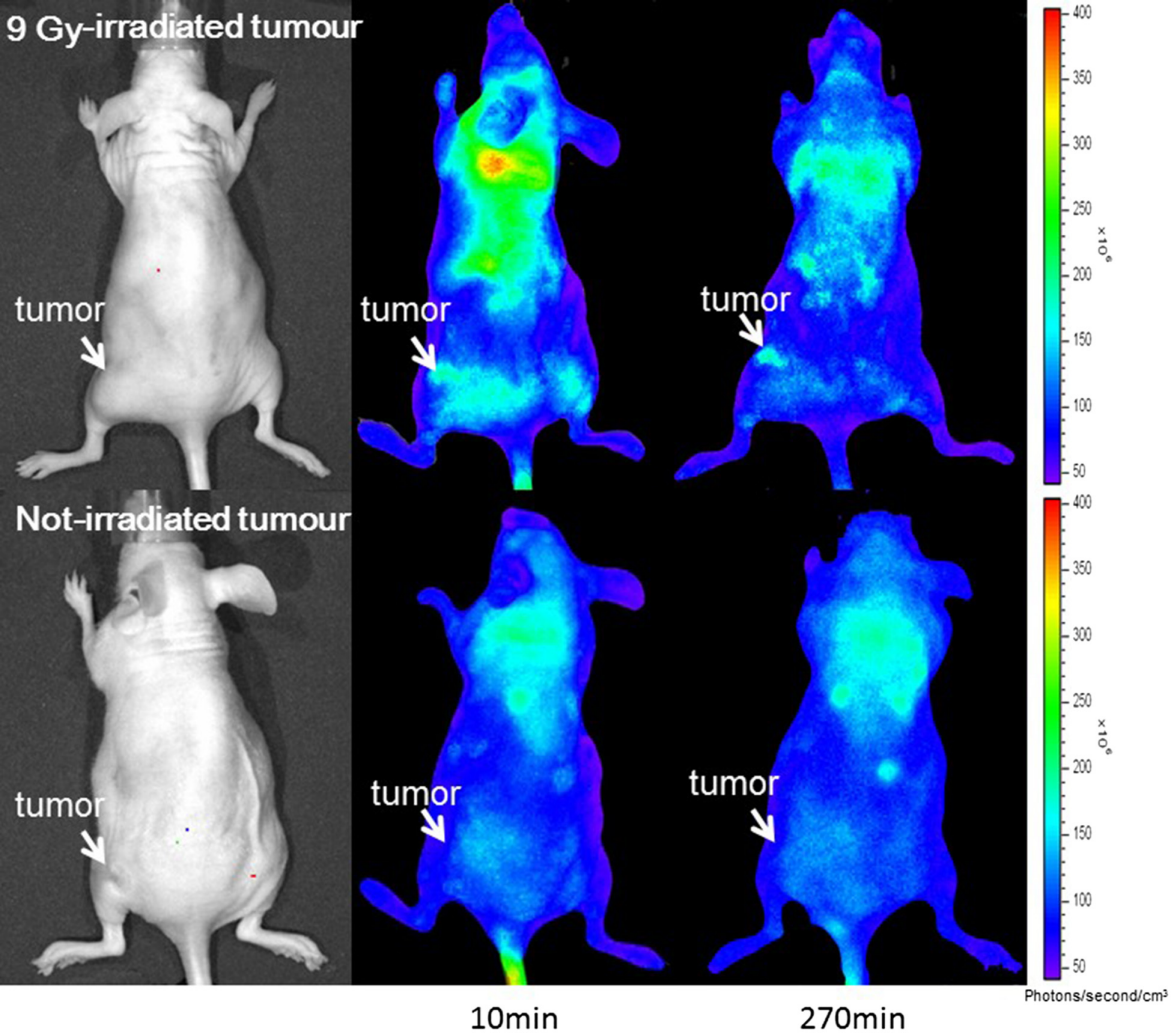
**Doxorubicin fluorescence imaging.** The upper section indicates an image of the 9-Gy-irradiated mouse. The lower section indicates an image of the not-irradiated mouse. The tumour site of the 9-Gy-irradiated mouse showed sufficient accumulation of doxorubicin in the upper section, whereas that of the not-irradiated mouse showed no significant accumulation of doxorubicin.

Figure 5 represents a fluorescence image of a xenograft. The tumour site of the 9-Gy-irradiated mouse exhibited fluorescence, indicating an accumulation of doxorubicin, whereas the tumour site of the not-irradiated mouse did not exhibit significant fluorescence, indicating little or no doxorubicin accumulation.

**Figure 6:**
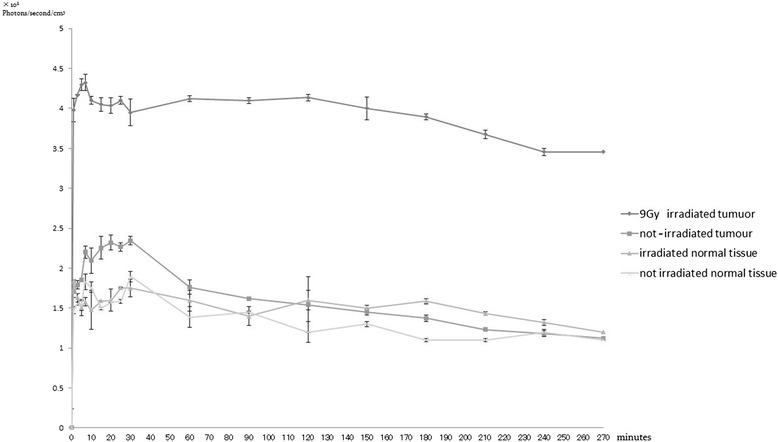
**Time-photon curve of doxorubicin in the 9**-**Gy-irradiated mouse and not-irradiated mouse.** The accumulation of doxorubicin in the tumour site of the 9-Gy-irradiated mouse was around 4 to 4.5 (10^8^ photons·s^−1^·cm^−3^) for approximately 120 min after administration and then washed out gradually. On the other hand, the accumulation of doxorubicin in the tumour site of the not-irradiated mouse was around 2 to 2.5(10^8^ photons·s^−1^·cm^−3^) at 30 min after administration, which was significantly lower than that in the 9-Gy-irradiated mouse. The site was subjected to washout at 30 min after administration, although the degree of washout was significantly greater than that observed in the 9-Gy-irradiated mouse.

Figure 6 shows the fluorescence photon count as a function of time. Compared to the tumour site of the not-irradiated mouse, the tumour site of the 9-Gy-irradiated mouse showed significantly higher accumulation, with rapid uptake over the initial 10 min after doxorubicin administration.

The washout rate for the tumour site of the 9-Gy-irradiated mouse was 4.3% ± 0.5%, whereas that of the tumour site of the not-irradiated mouse was 12.3% ± 2.2% by 30 min after doxorubicin administration. Approximately 120 min after doxorubicin administration, approaching the midpoint of photo counting, the washout rate of the tumour site of the 9-Gy-irradiated mouse was 3.4% ± 0.3%, whereas that of the tumour site of the not-irradiated mouse was 23.4% ± 3.5%. At 270 min after administration, i.e., at the endpoint of photon counting, the washout rate of the tumour site of the 9-Gy-irradiated mouse was 15.0% ± 2.3%, whereas that of the tumour site of the not-irradiated mouse was 45.8% ± 1.8%. Therefore, the washout rate of the tumour site of the 9-Gy-irradiated mouse was significantly lower, over the 270 min of measurement after administration, compared to that of the not-irradiated mouse, indicating that significantly more doxorubicin remained within the tumour.

### Cellular accumulation of ^99m^Tc MIBI in the presence of GG918 after irradiation

**Figure 7:**
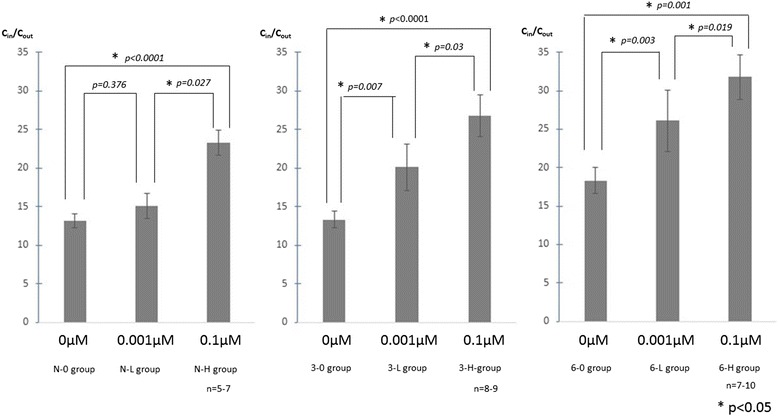
**Various concentrations of GG918 administered and accumulation of** ^**99m**^ **Tc MIBI in cells measured at 30 min after administration.** The *C*_in_/*C*_out_ at 30 min after the addition of ^99m^Tc MIBI is shown in the graph. The *C*_in_/*C*_out_ of the N-H, 3-H, and 6-H groups was significantly higher than that of the N-0, 3-0, and 6-0 groups. In addition, the *C*_in_/*C*_out_ in the N-H, 3-H, and 6-H groups was significantly higher than those in the N-L, 3-L, and 6-L groups. On the other hand, although there was no significant difference in the values between the N-0 group and the N-L group, those of the 3-L and 6-L groups were significantly higher than those of the 3-0 and 6-0 groups.

Figure 7 presents the accumulation of ^99m^Tc MIBI in cultured NSCLC cells to which the MDR modulator GG918 had been added. Although the ^99m^Tc MIBI accumulation in the N-H group was significantly higher than that in the N-0 and N-L groups, no significant differences in the accumulation between the N-L group and N-0 group were observed. ^99m^Tc MIBI accumulation in the 3-H group was significantly higher than that in the 3-0 and 3-L groups. ^99m^Tc MIBI accumulation in the 3-L group was significantly higher than that in the 3-0 group. The 6-0, 6-L, and 6-H groups also showed the same significant tendency as the 3-0, 3-L, and 3-H groups. ^99m^Tc MIBI accumulation was enhanced by irradiation, even in the presence of a low dose of GG918.

## Discussion

Pgp and BCRP are known to actively transport anticancer drugs, such as doxorubicin and cisplatin, out of cells; however, their actual mechanism of action remains elusive. This mechanism of active elimination of anticancer drugs is one key factor that gives rise to MDR. Reducing MDR transport function has been an important ongoing challenge in cancer treatment and has thus been extensively investigated [3,8,9]. This study revealed enhanced uptake of ^99m^Tc MIBI in NSCLC cells *in vitro* after 6- and 9-Gy irradiation, and an extended intracellular doxorubicin residence time in *in vivo* xenografted NSCLC tumours at 48 h after 9-Gy irradiation, although there was no difference in the expression of Pgp and BCRP between cells before and after 9-Gy irradiation immunocytochemically. Thereby suggesting that MDR transport function may have been reduced in both *in vitro* and *in vivo* experiments. In clinical lung cancer treatment, however, 6 Gy, 9 Gy, or higher doses of irradiation per radiotherapy do not constitute an established treatment method, due to a lack of evidence that reduces tumour volume. However, studies have shown that a reduction in MDR transport function can be achieved using low-dose fractionated radiation therapy (LDFRT) [18,19]. A previous study has demonstrated a reduction of MDR transport function in highly radiosensitive oral squamous cell carcinoma cell lines by 48 or 72 h after irradiation [18]. The administration of chemoradiotherapy, in combination with cisplatin, to NSCLC cells has also resulted in improved therapeutic efficacy of LDFRT, as compared to single-fraction therapy [19]. These reports suggested that the choice of irradiation method (e.g., LDFRT) and the time elapsed after irradiation, rather than the total radiation dose during treatment, may lead to a reduction in MDR transport function. The present study found no significant reduction in MDR transport function at 24 h after 6- and 9-Gy irradiation; *in vitro*^99m^Tc MIBI accumulation and *in vivo* doxorubicin uptake in xenografted tumours were both found to be non-significant (data not shown). However, at 48 h after irradiation, the *in vitro* accumulation of ^99m^Tc MIBI and *in vivo* doxorubicin uptake in the xenografted tumours were both significantly higher than in the not-irradiated groups. These results indicate the potential clinical utility of 6 Gy or higher doses of single-fraction radiation, which is contrary to the findings of previous studies. Although no report has demonstrated the efficacy of single-fraction radiation, we suggest that one possible reason for this was that the experiments were conducted at less than 48 h after irradiation.

Pgp and BCRP are localized in the cell membranes of normal cells, such as those of the intestines, lungs, proximal tubules of the kidney, and in capillary endothelial cells of the blood-brain barrier [20]. These transporter proteins are also present in the cells of lung and breast tumours. ^99m^Tc MIBI has long been used for imaging of myocardial perfusion and parathyroid gland uptake and is known as a substrate of Pgp [5,10]. ^99m^Tc MIBI thus exhibits the same kinetics *in vivo* as doxorubicin and cisplatin. Knowledge of the kinetics of ^99m^Tc MIBI and doxorubicin may help in planning treatment strategies for cancer. Doxorubicin accumulation in tumour cells can be monitored through fluorescence imaging if the tumour is located on the skin surface. Consequently, it is generally difficult to image doxorubicin as a tracer in humans, and thus, no previous studies have reported this specific imaging approach. Previous studies have shown that ^99m^Tc MIBI is a suitable radiotracer for imaging and can be used to image tumours [11,12]. Several reports using this approach have noted that the use of high-dose low-efficacy chemotherapy, with the associated serious side effects, can be reduced [5,21,22]. In addition, other reports have found that ^99m^Tc MIBI scintigraphy can be used for chemotherapy prognosis in lung cancer, wherein MRP, Pgp, or BCRP are expressed [23,24]. The present study also supported these findings and has shown that ^99m^Tc MIBI and doxorubicin appear to have similar kinetics.

In this study, we also investigated chemical modulators that reduce MDR transport function as well as the effect of irradiation on MDR transport function. Previous *in vitro* and *in vivo* studies have demonstrated that the mechanisms of action of the MDR modulators verapamil, GG918, cyclosporin A, and PSC833 depend largely on the dose of the compounds used [3,25]. GG918, a selective MDR modulator of Pgp and BCRP, inhibits the function of these transporters and increases ^99m^Tc MIBI cellular accumulation [10]. A phase I clinical trial using GG918 revealed problems relating to clinical safety and efficacy of this agent [9]. It has also been reported that a concentration of 0.01 μM GG918 is ineffective as a modulator and that MDR transport function may be reduced with high doses (0.1 μM or 0.2 μM); these concentrations, however, are toxic to humans [17,26]. In the present study, ^99m^Tc MIBI uptake in *in vitro* NSCLC cells was high after addition of 0.1 μM GG918 in the absence of irradiation. The ^99m^Tc MIBI uptake in NSCLC cells *in vitro* did not increase after addition of 0.001 μM GG918; however, after 3- or 6-Gy irradiation, the cellular uptake of ^99m^Tc MIBI significantly increased even after addition of only 0.001 μM GG918. These results suggest that the combination of irradiation and a low-dose MDR modulator may synergistically reduce MDR transport function.

## Conclusions

The NSCLC cell line H1299/wtp53 showed a reduction in MDR transport function after irradiation at a dose of 6 Gy or higher. The combination of a low-dose (3 and 6 Gy) irradiation and a low-dose MDR modulator, GG918, resulted in a synergistic effect that reduced MDR transport function.

## Abbreviations

^99m^Tc MIBI: technetium-99 metastable methoxyisobutylisonitrile
BCRP: breast cancer-resistant protein
LDFRT: low-dose fractionated radiation therapy
MDR: multidrug resistance
NSCLC: non-small cell lung cancer
Pgp: P-glycoprotein
